# Supermarket nutritionists’ perspectives, views, and experiences on affordability interventions to support healthier and more environmentally sustainable food purchasing in UK retail settings

**DOI:** 10.3389/fnut.2025.1710661

**Published:** 2025-12-16

**Authors:** Rebecca A Stone, Adrian Brown, Flora Douglas, Hannah Greatwood, Claire Griffiths, Emma Hunter, Alexandra Mary Johnstone, Marta Lonnie, Michelle Morris, Hannah Skeggs, Charlotte A. Hardman

**Affiliations:** 1 Department of Psychology, University of Liverpool, Liverpool, United Kingdom; 2 Obesity Institute, Leeds Beckett University, Leeds, United Kingdom; 3 Centre for Obesity Research, University College London, London, United Kingdom; 4 School of Health, Robert Gordon University, Aberdeen, United Kingdom; 5 The Rowett Institute, University of Aberdeen, Aberdeen, United Kingdom; 6 School of Food Science and Nutrition & Leeds Institute for Data Analytics, University of Leeds, Leeds, United Kingdom; 7 IGD, Hertfordshire, United Kingdom

**Keywords:** food insecurity, obesity, supermarket, affordability, healthy diet, sustainability, commercial, qualitative

## Abstract

**Introduction:**

Food insecurity (lack of reliable access to affordable and nutritious food) is a major concern in high-income countries because it increases the risk of poor nutrition, obesity and associated adverse health outcomes. Healthier diets are often also more environmentally sustainable (hereafter; sustainable), an important factor in reducing climate change. Practice-based interventions are therefore urgently needed to support people living with food insecurity and obesity to access and afford healthier and sustainable foods. Supermarkets are a key area for intervention, as purchasing can be an antecedent to consumption. However, the retailers’ perspectives on the feasibility of implementing affordability interventions is often overlooked and under-researched. Therefore, this study explored the perspectives, views, and experiences of major UK supermarket senior nutritionists on the acceptability and feasibility of using affordability interventions for healthier and sustainable food in the supermarket.

**Methods:**

We recruited seven UK senior supermarket nutritionists who represented 85% of the UK grocery market share. We used semi-structured interviews and analysed the data using a reflective thematic analysis approach.

**Results:**

Supermarket nutritionists perceived that their business did prioritise health and environmental sustainability for customers. However, there were several challenges encountered when trying to promote healthier and more sustainable food in the supermarket environment, including profitability concerns, unpredictability of intervention outcomes, control over own-brand products, perceived intention-behaviour gap, and a belief that they are already implementing affordability interventions. Differences in how supermarkets approach the evaluation of interventions also emerged, as well as a willingness to collaborate with academics and other retailers to optimise the evaluation of interventions. Lastly, supermarket nutritionists raised the need for an operationalised definition for sustainable food products.

**Discussion:**

Affordability interventions to support customers to purchase healthier and more sustainable food require supermarkets to consider multiple challenges. Findings highlight the need for upstream intervention that mandates and facilitates multi-lever approaches to health and sustainability without compromising commercial viability, along with practice-based approaches to implementation and evaluation.

## 1 Introduction

Across the globe, current dietary patterns are suboptimal for human and planetary health. In 2022, approximately 35% of the world population were unable to afford a healthy diet (1), contributing to poor quality dietary patterns that contribute heavily to the global burden of disease (2). Indeed, poor diet quality is directly linked to an increased burden of disease, including an increased risk of non-communicable diseases, such as stroke, cancer, heart disease, and is ultimately associated with overweight and obesity (3). Moreover, globally, a third of total anthropogenic greenhouse gas emissions (GHGE) are produced by the food system (4). Therefore, there is broad consensus that global dietary patterns need to be shifted in favour of health and environmental sustainability (1).

In the United Kingdom (UK), the government uses ‘the Eatwell Guide’ (consisting of nine recommendations) as a public health tool to define dietary recommendations on which foods and drinks constitute a healthy, balanced diet (5). As well as being optimal for human health, consuming a diet in line with the Eatwell Guide has been found to be environmentally sustainable [hereafter defined as sustainable—i.e., lower in GHGE, land use, and water use (6)]. However, according to an assessment of multiple observational cohort studies, adherence to all nine Eatwell Guide recommendations in the UK has been estimated at less than 0.1% (7), suggesting that the UK population’s diet is not meeting the requirements to promote population or planetary health. In England, obesity levels are a major public health issue, with approximately 68% of men and 60% of women classified as living with overweight, among whom 27% of men and 29% of women are classified as living with obesity (8). The drivers of obesity are complex, but often the food environment (i.e., the physical, economic, political, and sociocultural context that can influence an individual’s food choice) is implicated (9). Inequities within the food environment have also been highlighted, particularly for those living with low incomes as they can lack access to good-quality food (10, 11). In high income countries, people living in areas of high deprivation have a greater likelihood of living with obesity (12). One potential reason for this disparity is the experience of food insecurity (FI). FI is known as ‘the state of being without reliable access to enough affordable and nutritious food’ (13), and those who are food insecure are also more likely to be living with obesity (14, 15). Moreover, in our previous work we found that recent economic crises, such as the cost-of-living crisis (i.e., cost of everyday essentials rising more quickly than wages), have exacerbated the experience of being food insecure in people living with obesity (PLWO) and also been associated with greater use of specific food purchasing behaviours (i.e., use of budgeting, use of supermarket offers) and food preparation practices [i.e., use of energy-saving appliances, use of resourceful cooking styles (e.g., reducing food waste)] (16). Therefore, obesity and FI often co-occur, and the drivers of this relationship are likely exacerbated by recent global economic hardships (15), yet how best to support this group to purchase healthier and more sustainable food, within the complex food system, is poorly understood.

The food environment is one major component of the food system where dietary habits can be influenced, specifically within the retail food environment (17). In the UK, it has been estimated that 76–83% of food for consumption at home is purchased in a supermarket (18), depending on household income (19). Therefore, supermarket retailers are in a strong position (albeit not entirely responsible) to help encourage and enable customers to make healthier and more sustainable food purchases. Supermarkets can encourage customers by using interventions using behavioural levers from the traditional marketing mix. The four key levers are: placement (e.g., location of product in-store), product (e.g., range of products), promotion (e.g., offers and advertising) and price (e.g., baseline price of a product) (20–22). In a previous systematic review, which evaluated the effectiveness of supermarket interventions in their ability to change food purchasing at a population level, interventions were categorised as ‘economic’ (i.e., price increase, decrease, or financial reward), ‘store environment’ (i.e., changes to microenvironment), or ‘labelling/ education’ (i.e., product labelling or customer education) (23). Results from this review indicated that economic interventions were the most effective for influencing purchasing behaviour towards healthier purchases.

UK supermarkets have publicly committed to supporting customers during the cost-of-living crisis (24) and often implement and evaluate interventions to promote healthy and sustainable food purchasing (25, 26). For example, national supermarket chain Sainsbury’s plc implemented a targeted supermarket intervention that was aimed at supporting customers with Healthy Start vouchers, by offering an additional £2 top-up voucher to this scheme. The Healthy Start scheme (27) is a government initiative targeted at low-income pregnant mothers (10 weeks into their pregnancy) and parents/caregivers who are responsible for at least one child under 4-years of age. The scheme provides vouchers towards the purchase of fruit, vegetables, pulses, milk, or infant formula. Findings from the £2 top-up scheme indicated that customers purchased on average 13 times more portions of fruit and vegetables per transaction, and their purchasing reflected greater adherence to the Eatwell Guide (28). They also purchased fewer meat and discretionary products (29). These results indicate that supermarkets are a key sector that could be optimised to better enable vulnerable groups to purchase healthier and more sustainable food products.

In a previous study (30), our research team gathered insights from PLWO about what supermarket interventions they would find most helpful to support them in purchasing healthier and more sustainable food in the supermarket. Our sample (*N* = 583) indicated that interventions relating to price and incentivisation [i.e., economic interventions, as per categorisation in Hartmann-Boyce et al. (23) would be the most helpful, and interventions relating to labelling/education would be the least helpful]. These findings were complemented further by our parallel qualitative work through in-depth interviews with PLWO and FI, who highlighted the severity of living with financial constraints with regards to shopping for healthy and sustainable food in the supermarket (31). Therefore, employing supermarket interventions that are related to price and affordability may be pivotal in addressing dietary inequalities in PLWO and FI.

Whilst research indicates that in-store affordability interventions can benefit healthier food purchasing (32), the feasibility of implementing these interventions from a commercial perspective remains unclear (33). Existing studies highlight the significant barriers faced by retailers, including profitability concerns and limited consumer demand, which are associated with promoting healthier food (34). However, such studies are either in the context of small stores or independent supermarkets that do not reflect a large grocery market share and scalability of intervention was not tested. Therefore, the current study sought to address this evidence gap by using a qualitative methodology to explore the perspectives, views, and experiences of major UK supermarket senior nutritionists on the acceptability and feasibility of using price and incentivisation (hereafter defined as ‘affordability interventions’) as interventions for healthier and more sustainable purchasing in the supermarket setting.

## 2 Methods

### 2.1 Design

We conducted a qualitative study using semi-structured online interviews with a purposive sample of seven senior supermarket nutritionists from major UK supermarket retailers. Supermarket nutritionists were identified through a gatekeeper organisation, IGD (Institute of Grocery Distribution), who provided access to an existing, established strategy group of senior nutritionists from leading retailers and manufacturers. Within this group, there were 10 senior supermarket nutritionists (each representing a single retailer) who collectively represented 98% of the UK grocery market share (35). During a routine strategy group meeting, the study was advertised by the IGD (using material co-designed with the researchers) and expressions of interest from supermarket nutritionists to participate in the study were shared with the academic lead. Seven of the 10 supermarket nutritionists agreed to participate, representing retailers with 85% of the UK grocery market share (35). The recruited sample represented discounters through to higher end supermarket retailers. Due to the sensitive nature of UK competition law, according to the Competition Act (1998), the strictest confidence was employed during the interviews to ensure retailers remained anonymous. Participating retailers were provided with a participant information sheet, the interview schedule, and a consent form prior to the interview. Written consent was obtained in advance of the interview. No compensation was given to the participating retailers. Ethical approval was granted from the University of Liverpool Research Ethics Committee, Ethics number 12622.

The interview schedule was co-produced in partnership with the wider project team [members of FIO Food (36)], with our industry retail partner (a major UK supermarket), and a social impact organisation (working alongside the food and grocery industry undertaking research for the benefit of the public). A copy of the interview schedule is in Supplementary materials 1. Notably, the interview guide began by asking respondents how their supermarket defined a ‘healthy’ food product and a ‘sustainable’ food product. These questions were designed to elicit respondent’s operational definitions rather than impose predefined criteria. As captured in sub-theme 4.1, respondents ubiquitously referred to the UK’s Nutrient Profiling Model (37), or metrics that use the Nutrient Profiling Model (i.e., high in fat, sugar, and salt (HFSS) status). In contrast, due to the absence of a legally recognised definition for a sustainable food product, respondents were encouraged to draw on whichever environmental or social dimensions were salient within their supermarket.

The interviews were conducted online by RAS between August 2023–October 2023, one to one, using Microsoft Teams and lasted approximately 30 min (range: 19–41 min). During each interview, the participant gave additional verbal consent. Each interview was video recorded using Microsoft Teams’ recording feature. Interviews were transcribed verbatim using Microsoft Teams’ auto transcription feature into Microsoft Word within 24 h of the interview. RAS manually checked all auto transcriptions for accuracy. The video recording of the interview was deleted immediately after transcription. RAS anonymised all transcripts and assigned pseudonyms for each supermarket (e.g., Supermarket A). The anonymised transcripts were then sent to the corresponding supermarket for their approval regarding the level of anonymisation and for sense-checking. As a further privacy step, after analysis, two of the project team were provided with a full list of anonymised retailer quotes to assess whether supermarkets were identifiable. All quotes were deemed non-identifiable.

### 2.2 Data analysis

The transcripts were thematically analysed by two of the authors (RAS and CAH) following steps outlined by Braun and Clarke (35). The analysis was conducted using an inductive ‘bottom-up’ approach in which there was no attempt to fit the data into an existing theory (38). The steps involved, (i) *familiarisation*; reading each transcript completely to familiarise with the data (RAS), (ii) *coding*; generating initial descriptive codes for relevant information in each data item (RAS), (iii) *initial themes*; exploring relationships between codes to form a structure (RAS, CAH), (iv) *developing and reviewing themes*; relationships between codes that formed themes were reviewed and discussed until consensus was reached (RAS, CAH, and wider FIO Food project team during routine research meetings), (v) *naming and defining themes*; theme names and their definition were derived from the meaning of the clustered codes (RAS and CAH). *NVivo 14* software was used to manage and support data analyses.

## 3 Results

### 3.1 Thematic analysis

Four themes were generated inductively from the reflective thematic analysis with 12 associated subthemes (Table 1). Each theme is defined and then the subthemes are described with various retailer quotes to illustrate findings.

**Table 1:** Summary of themes and subthemes following thematic analysis of retailer interviews (*N* = 7).

Theme	Subtheme
Business pledge: Supermarkets’ Commitment to Health and Sustainability	Health and Sustainability Embedded in Supermarket Identity Supermarkets’ Ethical Obligations to Health and Sustainability Strategic Alignment with Health and Sustainability
Navigating complexities in healthy food promotion in the supermarket	The “We do this Already” Gap Balancing Act: Profitability vs. Healthy Food Promotion Expecting the Unexpected: Mismatched Intervention Effects and Competing Messages in Supermarkets “We have the Biggest Influence Over Them”: Control Over Own Brand Products Perceived Customer Ambivalence: The Challenge of Healthy Food Demand
Evaluation Challenges and Pursuing Collaborative Solutions	Illuminating Supermarket Blind Spots: Limitations in Evaluation and Reporting Collaborating Towards a Healthier, and more Sustainable Future
Navigating the Undefined: Challenges in Promoting Sustainable Food Choices	The Complexities of Defining and Promoting Sustainable Food Choices Estimating Sustainability through Accredited Sourcing and Proxy Measures

### 3.2 Theme 1: “business pledge: supermarkets’ commitment to health and sustainability”

When asked about their supermarkets’ approach to health and sustainability, respondents universally claimed that their businesses had an “*aligned vision*,” which mostly spanned the entire business and was navigated by top level management (albeit for some this was a recent interest from top level management and a recent convergence of health and sustainability strategies). Moreover, the majority of respondents referred to their supermarkets’ approach to health and sustainability as “*embedded within their supermarket’s identity*,” where supermarkets believed they had corporate “*responsibility*” for ensuring both the customer’s health and planetary health, due to the powerful role that they play in shaping customer purchasing behaviour.

#### 3.2.1 Subtheme 1.1: “health and sustainability embedded in supermarket identity”

Central to this subtheme is the shared belief across respondents that the healthiness and/or sustainability of food for customers are integral components of their DNA, reflecting a deeply ingrained and inflexible commitment to these values. As exemplified in the following quote, respondents felt that their organisational ethos and guiding principles were governed by these components:

*“So, we've got a rich legacy really. So, we were one of the first retailers to have a healthy living brand—so health has always been a part of our DNA.”* (Supermarket E)

#### 3.2.2 Subtheme 1.2: “supermarkets’ ethical obligations to health and sustainability”

This subtheme refers to respondents’ acknowledgment of their ethical responsibility toward both customer health and planetary well-being, which aligns with the concept of corporate social responsibility. Indeed, in the following quote this respondent recognised their position as a powerful actor in the food system:

*“I guess the case with sustainability is that we, as a food industry and as a big retailer, have our part to play in helping to ensure that we have a sustainable future for everyone.”* (Supermarket A)

Moreover, the collective importance placed on corporate social responsibility in guiding supermarkets’ decisions and actions may reflect supermarkets’ proactive stance in contributing positively to society and the environment. For example, Supermarket E said:

*“We know that our business depends on the world around us. As a UK retailer, we know we can make a big difference. Our commitment to operating in a responsible and sustainable way reflect our values.”* (Supermarket E)

Respondents reported that supermarkets conceived their role in promoting healthy, sustainable diets as integral to supporting customer health and planetary health. This emphasizes how supermarkets’ ethical obligations are equally as imperative as other business outcomes, such as profit motives:

*“So yeah, the strategy is grounded within accepting and acknowledging that as a food retailer we play a really important part in making sure that healthy eating is exciting and easy.”* (Supermarket B)

However, whilst appearing philanthropic, supermarkets corporate social responsibility behaviours appeared to be related to the wider pressures of being seen to be doing *“the right thing,”* such as from social pressure. This sentiment is illustrated by the following two respondents:

*“And there is an element of ‘you have to do the right thing.”* (Supermarket C)

*“We've also made [name of low-cost pricing strategy for healthy food], partly because it's the right thing to do.”* (Supermarket G)

#### 3.2.3 Subtheme 1.3: “strategic alignment with health and sustainability”

All respondents flagged the strategic commitment of their supermarket to prioritise both health and sustainability initiatives at the highest levels of leadership. For example:

*“So, the responsibility to achieve commitments/strategy on health and sustainability lies at manager level, director level. Higher level management is highly invested in achieving these commitments too. Supermarket F is a close family, and we take our commitments very seriously and we all are accountable.”* (Supermarket F)

However, for some respondents, the involvement of the highest levels of leadership in sustainability specifically had only occurred recently, suggesting that there has been a pivot in the priorities of supermarkets:

*“So, the top is really engaged in the Environmental, Social, and Governance (ESG) now, but that's literally been agreed in the past [period of time].”* (Supermarket C)

Indeed, the prioritisation of sustainability may have resulted from external pressures, such as growing customer concern for climate change, or pressure from Non-Governmental Organisations (NGO’s) to make pledges in line with their initiative (e.g., World Wildlife Fund (WWF) Livewell Basket (39) campaign, where supermarkets pledged support to the ambition of halving the environmental impact of UK shopping baskets by 2020), as illustrated by the following respondent’s quote:

*Interviewee: “I think we've been doing various initiatives, but I would say of the last three years, the [name of policy] programme came into force, and it was a change being led by the board…”*

*Interviewer: “and what do you think brought about that change?”*

*Interviewee: “… I think you would be wrong to shy away from you know NGO pressure and what's happening in market.”* (Supermarket G)

Nevertheless, most respondents noted that their strategy for health and sustainability operated as a combined, dual approach, rather than individually, which suggests that supermarkets are beginning to appreciate the intersectionality of health and sustainability and the idea that it exists as part of a system. This streamlined approach to health and sustainability was seen by respondents as being supported by a range of departments and made use of branded (i.e., named) roadmaps/strategies to drive meaningful change across their operations:

*“So basically, the overall strategy is called [name of policy] and basically healthy and sustainable diets are a pillar of one of those work streams.”* (Supermarket G)

*“So, we have a sustainability strategy that would cover health in it. So, in [year] we launched [name of commitment/policy for supermarket D] which is Supermarket D’s how we intend on being sustainable until [year]. Supermarket D’s commitments for a healthier future sit within this plan.”* (Supermarket D)

As illustrated in the following quotes, it was discussed by some respondents that their supermarket uses a dual strategy for health and sustainability, rather than individual. Respondents suggested that this was because of an increasing customer awareness of the interconnectivity between health and sustainability which had not been observed in the market before. This emphasises the key role that customers play in shaping supermarkets’ approach to business:

*“Well, years ago we've done them [health and sustainability interventions] separately, so we had health events where when you go down the ‘power aisle’ and it was all about health messaging. But, I think customers are starting to see the two pieces (health and sustainability) coming together a little bit more as time goes on, and by living in the environment that they're in.”* (Supermarket E)

*“Especially over the last five years or so as it's imperative to be choosing healthy diets, you've got that broader "good for me" and "good for the planet" motivation that customers have. I think our strategy sits very well alongside that.”* (Supermarket B)

### 3.3 Theme 2: navigating complexities in healthy food promotion in the supermarket

When discussing their historic experiences of using affordability interventions to promote healthy food in the supermarket, most respondents recounted various challenges that they had encountered. Due to these challenges, supermarkets indicated that future healthy food promotion interventions would require greater assurances that interventions would operate optimally (i.e., as hypothesised). For some respondents, they questioned the proposal that supermarkets should use more affordability interventions to support PLWO and FI given that they believed *“they were already*” implementing sufficient affordability interventions. For other respondents, they highlighted the difficulty of promoting healthy food over HFSS food, as promoting the former was considered *“margin dilutive”* (decrease in profits). Most respondents also flagged the unpredictability of customer behaviour regarding healthier food promotion, where investing in affordability interventions can result in contrary effects to the hypothesised outcome (e.g., no increase in sales for the promoted product). It was suggested that this could be a result of the effect of “*competing messaging in store*” and the “*influence supermarkets have over branded and own brand products.”* Alternatively, it could reflect the ‘say-do’ gap, where despite *“fulfilling customer demands for healthy food promotion, this does not always result in an increase in sales.”*

#### 3.3.1 Subtheme 2.1: the “we do this already” gap

When discussing the proposal to implement more supermarket interventions relating to affordability, one respondent expressed confusion as they believed they already implemented such interventions. They questioned how PLWO and FI were interpreting these interventions, which highlights a potential gap between customer experiences and existing practices:

*“To be honest when I was reading this question, I was thinking "we're already doing this and most retailers are, so it made me question what are the participants classing as healthy? Why do they think that we're not doing this? Is it something we need to do more of?”* (Supermarket D)

Similarly, a respondent from another supermarket showed an awareness of what interventions their competitor supermarkets had historically implemented. They also indicated that affordability interventions are, currently being implemented or have been implemented previously:

*“I think either people have done them before or they're doing them [price/ incentivization and store environment interventions].”* (Supermarket E)

#### 3.3.2 Subtheme 2.2: “balancing act: profitability vs. healthy food promotion”

Respondents frequently mentioned the commercial viability of healthy food whereby there were clear differences in profitability between that of healthy food (often own branded) and of unhealthy food (often branded). One respondent referred to differences in profitability between unhealthy food (i.e., crisps and chocolate in this case) compared to that of healthier food due to customer popularity. They indicated that sales of healthier food were naturally lower than less healthy food and so promoting healthier food products was not commercially sensible as the intervention would not likely increase sales:

*“You know there's a commercial element around what's commercially viable because healthier food is less profitable, and I know that's a really broad statement, but if you think about the categories where you've got unhealthy food like crisps and chocolate, the healthier versions of those products are not as popular with customers and they're not as profitable.”* (Supermarket C).

Similarly, another respondent underscored their awareness and desire to support customers but stressed how this desire must be balanced with remaining a commercially viable business.

*“In terms of price, If I'm being completely honest, in terms of inflationary pressures, it's a commercial business, it's really, really tricky to do something different…I think price is probably the most important thing to our customers, particularly in the cost-of-living crisis, but it's probably the hardest one to do from a business perspective.”* (Supermarket G)

Two respondents took this challenge further and indicated that whilst it would be ideal to promote healthier food, advertising budgets are limited and so without seeing an uplift in sales of the promoted food, the pricing investment must end. This is likely to partly be the result of healthier food (i.e., fruit and vegetables) being an own-brand product, meaning that affordability interventions are funded purely by the supermarket, not with the support of suppliers’ funding:

*“I suppose if we did invest money in a [healthy] product, I don't know how long we would need to keep it on for before you would need that investment to go on to another product. I suppose businesses only have so much money, so they can't keep pumping money into price investment.”* (Supermarket D)

As a result, respondents highlighted the difficult decision that must be made between funding an intervention in favour of health (which is often own branded, like fruit and vegetables), against promoting branded, products (often HFSS) where sales are more likely to be made:

*“And then when it's an own brand product, like produce (i.e., fruit and vegetables). They’re already, you know, maybe margin dilutive (i.e., decreases profit margins) or might not have profit. So, where does the money come from to drive those promotions to promote them on an end where you could be making money from a branded product?”* (Supermarket C)

Indeed, another respondent poignantly highlighted that any interventions related to the price of healthier products must be profitable as supermarkets are not *“charitable”:*

*“A key element of our strategy that we're putting forward, our ambition, is to make it profitable and sustainable to make healthy choices accessible, because otherwise we can't do it because we are a business, it can't be a charitable intervention because you can only support that for a period of time.”* (Supermarket C)

#### 3.3.3 Subtheme 2.3: “expecting the unexpected: mismatched intervention effects and competing messages in supermarkets”

Whilst there was uncertainty regarding the profitability of promoting healthier food, a further barrier that was mentioned related to the unpredictability of intervention outcomes due to the store environment. There was broad agreement between respondents that the store environment was a complex arena with many activations happening simultaneously. As a result, interventions that are theoretically sound can occasionally result in unexpected outcomes or even failure, as it is not possible to solely implement one intervention in isolation in a real-world supermarket. For example:

*“I think if anything, what we are seeing is that these hypotheses are one thing, but what happens in real life is quite another, especially when there's so many other things going on in the store.”* (Supermarket A)

One respondent recalled their experience of an intervention that was aimed at supporting customers to transform their baskets in favour of health. However, despite being well-intentioned, when implemented, there were other in-store marketing activations running simultaneously, specifically on seasonal products (i.e., “*alcohol and BBQ stuff*”). As a result, the respondent believed this hindered the successfulness of their health intervention, and even appeared to express a sense of defeatism by reducing their well-theorised intervention to “*just noise*.” Therefore, the competing messaging in-store may reflect an incongruence in business interests between supporting customer health and promoting sales of foods preferred in a season (which are often less healthy):

*“I think it's very difficult in a busy retail environment, you just can't influence what else is going on in the shop. We've run initiatives before where our customer said they wanted more help finding healthy diets, so, as a group of nutritionists, we all went into the shop and did healthy basket makeovers for customers. We were taking customers on healthy store tours showing them stuff too. But, during that, other stuff was going on in the shop at the same time, which undermined the message slightly. Like, a massive front of store packed full of alcohol and BBQ stuff…”* (Supermarket B)

Nevertheless, respondents’ awareness of this unpredictability appeared to have instilled a level of predictability, where they expect “*there are some circumstances that they cannot control*”:

*“Interventions might run coincidentally at the same time as a big event or something that's happened in the news, so, we've learned that there are some circumstances that we can't control.*” (Supermarket E)

#### 3.3.4 Subtheme 2.4: “we have the biggest influence over them”: control over own-brand products

Several respondents referred to the challenge of only being able to influence their own-brand products and as a result, existing health strategies were often limited to only these products. Another respondent highlighted that their supermarket’s health targets and commitments would only be related to own-brand products because this was the area where they were able to “influence” the most:

*“It [health targets/commitments] most likely would be on own brand stuff just because we have the biggest influence over them. So yeah, all of our own brand products would have to meet salt, calorie targets and use recyclable packaging, have fair trade ingredients.”* (Supermarket D)

The respondent went on to highlight how, as nutritionists, their power and influence over the supermarket environment was limited. Like the previous subtheme regarding the commercial viability of healthy food promotion and competing messaging in-store, the following respondent described how, in addition to other initiatives being promoted in-store (e.g., BBQ food in summer), suppliers and manufacturers of branded food can also obscure health intervention messages:

*“There's always lots of different messages, lots of different initiatives, even stuff I've got no control over, like things that suppliers come in and do, and that can make it quite tricky as well.*” (Supermarket B)

#### 3.3.5 Subtheme 2.5: perceived customer ambivalence: the challenge of healthy food demand

The price of healthier food was frequently mentioned by respondents as being their customers’ largest barrier to healthy eating. Thus, the suggestion to implement affordability interventions was unsurprising to our respondents, such as:

“*I'm not surprised customers have said price because obviously price is one of the key barriers to healthy eating.”* (Supermarket C)

However, respondents highlighted how when price is removed as a barrier to healthy eating (i.e., through affordability interventions), customers do not always increase their purchasing of healthy foods. Instead, one respondent suggested that the convenience of less healthy food was more alluring than the removal of price as a barrier to healthy food:

*“Whilst customers say that they want healthier choices, they don't necessarily shop in that way… And the issue is that even when you promote those healthier choices, it doesn't necessarily mean that customers buy more of it, because, often the healthiest choice is the least convenient, and you've got to do something to it to prep it, to make it easy to eat, and that takes effort.”* (Supermarket A)

Alternatively, another respondent attributed this ambivalence to customer preference, where the price and promotion of less healthy snacks (in this case, crisps) and its healthier counterpart (in this case, baked crisps) were held constant, sales of the less healthy snack were highest:

*“What we see in the data is even when they're the same price, people don't purchase them or don't purchase them as much…If you take baked crisps and normal crisps, I know they're not the healthiest thing, but it's a healthier alternative within the category. They're the same price and they're on the same promotion, but we always will sell more of the standard variant. So, it's not that price is the barrier in that instance, it's that customers prefer the less healthy product.”* (Supermarket C)

### 3.4 Theme 3: evaluation challenges and pursuing collaborative solutions

Throughout all interviews, respondents reflected on their own and their competitors’ use of affordability interventions (e.g., loyalty cards) to support customers with purchasing a healthier and/or more sustainable diet. Respondents described their approach to (or their lack of) evaluating the effectiveness of interventions, and this highlighted the “*varied and diverse approaches*” taken to intervention evaluation between retailers. Despite these differences, most respondents showed a prevailing *“willingness to collaborate*” with other retailers and academics to maximise the effectiveness of affordability interventions for health.

#### 3.4.1 Subtheme 3.1: illuminating supermarket blind spots: limitations in evaluation and reporting

Several respondents discussed the lack of standardisation between retailers in how supermarket interventions were evaluated and reported. Intervention evaluation appeared to exist on a continuum between respondents, where some were more advanced (such as respondents from retailers who assessed basket sales and commercial return: profitability, product margins, price elasticity) and others who were in their infancy. For those respondents from retailers in their infancy, this did not indicate that supermarkets were not implementing many different interventions, rather it reflected a potential shortfall in their approach to assessing the impact of those interventions:

“*We've done loads of stuff on health and sustainability over the years, but we've been really bad at evaluating it, so you know, we look to other retailers and we think people are doing a much better job of saying like “we did X and it had this impact.”* (Supermarket C)

One respondent spoke on behalf of all retailers *(“I think we are all in agreement”)* to underscore the volume of supermarket interventions for health in retail to date. They warned of the potential confusion experienced by retailers from the number of often repeated interventions for which outcomes and effectiveness may vary. They noted that a consequence of the lack of standardisation to intervention evaluation was the inability to take learnings from other retailer’s intervention efforts:

*“I think generally as retailers, I think we are all in agreement that actually there are so many trials going on, so, how do you work out what's different and what's new and what's really going to turn the dial?”* (Supermarket E)

Respondents described the repetition observed between supermarkets in their interventions to promote healthy food. They highlighted the expense associated with conducting a supermarket intervention and how this investment would be best spent “*more wisely*” Respondents highlighted the potential utility of a resource that assimilated findings from active/previous interventions and provided best-practice guidance for evaluation:

*“I think sometimes there isn't a clear, best practice guide out there of what we should be doing… There’re far too many people doing similar things. And actually, money could be used much more wisely to bring about the same amount of change.”* (Supermarket G)

#### 3.4.2 Subtheme 3.2: collaborating towards a healthier, more sustainable future

Several respondents indicated that they were willing to collaborate to share findings to achieve a healthier, more sustainable future. For instance:

*“And we're wanting to work with partners on that. So external partners and internal partners across the business to say where should we focus our investments, what are the right things [interventions] to be working on.”* (Supermarket C)

Another respondent noted the expense of conducting an intervention and the assumption that it will benefit both the customer and the business. Therefore, evaluating the impact of interventions and sharing findings across retailers would be greatly beneficial, both for the customer (to find “*what works*”), but also for the business (to “*reduce costs*” associated with ineffective interventions):

*“It's just learning what works really. It costs us money to run trials, but it would cost us even more if we were just to go out and do it permanently and it not work out. Even if we try something, it would be good to share it externally so we can see what works. But yeah, it’s just industry working together and sharing learnings.”* (Supermarket D)

One respondent referred to their partnership with academic researchers as a “*journey*,” and this suggests awareness that sharing intervention learnings cross-retailer will likely be enduring, complex, and not without its complications:

*“I suppose it's about bringing [supermarket] research and academic research and the right partners together to help us on that journey.”* (Supermarket G)

Together, this suggests how retailers are essentially onboard with the process of improving the status quo of intervention data sharing.

### 3.5 Theme 4: navigating the undefined: challenges in promoting sustainable food choices

Almost all respondents highlighted the barrier to promoting sustainable food products. They indicated that, although they were willing to conduct interventions in support of sustainable food purchasing, the “*lack of a legal definition*” for claiming a food product was sustainable was discouraging efforts. As sustainability was a relatively new prioritisation, the infrastructure to endorse a sustainable diet appears to have not yet been created. To remedy this lag, most respondents claimed they use existing, legally *“certified standards*” (e.g., Fair Trade) as a proxy for official definitions of sustainability.

#### 3.5.1 Subtheme 4.1: “the complexities of defining and promoting sustainable food choices”

As illustrated in the following quotes, there was a lack of an official, universal definition for what constitutes a sustainable food product, as opposed to a healthy food product where there are numerous guidelines (e.g., Nutrient Profiling Model (37), The Eatwell Guide):

*“Yeah, mainly I think we haven't got a definition. Unlike for health where we've got an own healthy brand logo, we haven't got an own brand sustainable logo.”* (Supermarket B)

Moreover, most respondents reflected on how the lack of an official definition had acted as a barrier to promoting foods as being a sustainable choice. Respondents recalled how other retailers had encountered fines from regulatory bodies [i.e., Advertising Standards Authority (ASA; UK’s independent regulator of advertising across all media)] for claims they had made regarding the sustainability of a food product. In the following quote, this respondent expressed a desire for tighter governance regarding the sustainability of food products so that they could promote products legally:

*“So, the ASA started to fine a lot of companies for making what they considered 'misleading claims'. Sometimes that was absolutely true, but I think there also just needs to be some really clear boundaries and kind of frameworks for us to work to so that we know when we're developing products and communications of what we can say and what we can't.”* (Supermarket A)

This quote, along with the following quote, conveys how there was a buy-in from supermarkets to transform customers’ baskets in favour of planetary health, but illustrates how supermarkets are constrained by the legality of supporting such changes and their wariness as a result. One respondent described how they had implemented interventions to increase the purchasing of food that could be viewed as both healthier and more sustainable (e.g., increase the sales of vegetarian sandwiches), but due to the lack of an official definition, they were unable to report their findings from a sustainability perspective:

*“I mean, we've definitely tried to have trials in healthy, sustainable food, but the problem is you can't talk about it so then where's the commercial benefit?”* (Supermarket C)

#### 3.5.2 Subtheme 4.2: “estimating sustainability through accredited sourcing and proxy measures”

Instead, as illustrated in the following quotes, respondents spoke of how they used a variety of legal “*shortcuts*” to define sustainable food products. For example, by using accredited sourcing and certified standards like Fair Trade and the Soil Association to legitimise one area relating to the sustainability of the product. However, as noted by the following respondent, these shortcuts were not considered a silver bullet and instead reflected a well-intentioned estimate using the available legal resources:

*“The best shortcuts that we have are things like certification schemes: Responsibly sourced? Fair trade? Because things like that, which are third-party organisations, validate one aspect of the sustainability of a product we could potentially use, but as I said, it's not without criticism that just because you are fairly traded doesn't necessarily mean you are, you know, environmentally sustainably sourced, in a recyclable packaging, for example. So, things like that can be a real issue.”* (Supermarket A)

## 4 Discussion

Using interviews from seven UK supermarket nutritionists, representing 85% of the grocery market share, this study explored perspectives, views, and experiences on the acceptability and feasibility of using affordability interventions to support the purchasing of healthier and more sustainable foods. We identified four themes and 12 sub-themes. The first theme suggested that there was the perception that supermarket retailers prioritise health and sustainability. However, the remaining three themes reflected the complexity of achieving this intention, as based on respondents’ experiences, there appeared to be several challenges that were encountered when trying to promote these foods, including profitability concerns, unpredictability of intervention outcomes, control over own-brand products versus branded, a perceived intention-behaviour gap, and a belief that supermarkets are doing these interventions already. Additionally, differences in how supermarkets approach intervention evaluation also emerged, and a need for an operationalised definition for sustainable food products.

Every respondent adopted the view that their businesses’ approach to health and sustainability was “*embedded in their identity*” as a supermarket. This perception is at odds with public perceptions of supermarkets (and the food industry in general) where they are often viewed as large contributors to disease and ill-health (40, 41). Research indicates that supermarkets show a price-promotion (i.e., total price reductions and multi-buy) bias towards less healthy foods [i.e., HFSS (42)], where these price-promotions encourage additional purchases and greater purchase volumes of less healthy food. However, despite supermarkets’ apparent health-halo, our findings do align with previous literature who argue that supermarkets have a split corporate responsibility to health and sustainability (43); whilst strategies used by supermarkets may contribute to public health problems, they are simultaneously engaging in activities to prevent them.

Activities that are implemented by supermarkets to prevent public health problems are an example of supermarket’s approach to Corporate Social Responsibility (CSR). CSR approaches differ based on corporate motivations; they can be viewed as either instrumental (i.e., CSR to generate profit), ethical (i.e., CSR is an ethical obligation to society), integrative (i.e., supermarkets rely on society and so CSR is needed to support continued success), or political (i.e., supermarkets possess a degree of power that demands they act responsibly for their CSR) (44, 45). In our study, supermarkets’ approach to CSR appeared strongly aligned with ethical and political theories, as they have an “*ethical obligation*” for their customers in relation to health and sustainability, which in part is based on being cognisant of the power they possess in the food industry. This finding contrasts with previous work, where in a qualitative study of Danish supermarket managers, supermarket staff assigned the main responsibility for healthy living on the individual customer (33). Instead, our findings align with those who have shown most retailers display (publicly espouse) responsibility for the health of their community and customers (34, 46). However, in Martinez et al. (32) they also report that supermarkets may promote healthier food to generate profit (i.e., instrumental theory), which differs with our respondents’ belief that their supermarkets’ approach to health and sustainability are ethically based. It is possible that this discrepancy is attributed to the fact that only supermarket nutritionists provided their perspectives in our sample. In comparison, Martinez et al. (32) analysed a variety of perspectives from different positions across the supermarket, including corporate managers whose view might differ.

An important finding from our work relates to the number of complexities associated with promoting healthier food in the supermarket. Our previous work with the lived experience of PLWO and FI advocates for more interventions based on price and incentivisation (30). However, the current findings suggest that this recommendation does not acknowledge the barriers to implementation that are encountered by supermarkets, from the perspective of a supermarket nutritionist. Firstly, we were interested to note the strongly held belief of most respondents that supermarkets were already *“do[ing] affordability interventions”* on healthier food, such as loyalty card pricing. This finding also complements other research where supermarket retailers have described, from their perspective, that consumers were already provided with many opportunities to make healthier food choices (33). However, our previous work suggests this finding contrasts with the views of customers. For example, Hunter et al. (31) highlighted the difficulties faced by PLWO and FI with using existing affordability interventions in supermarkets, such as how deals on foods fluctuate. Together our findings suggest that current affordability interventions to support the purchasing of healthier food do not resonate with the intended beneficiaries of affordability interventions and this disconnect merits further exploration in future supermarket intervention development.

Another challenge that was mentioned by our respondents was the viability of healthier food promotion from a commercial perspective. Almost all respondents (*n* = 6) highlighted the low profit margins that were associated with healthier food promotion in comparison with promoting less healthy products. Indeed, previous literature has highlighted how return on investment is a central consideration to executives when employing an intervention (47). Our findings underscore the contentious conundrum that is faced by food retailers, from the perspective of supermarket nutritionists, between wanting to provide customers with healthier food using affordability promotions, whilst also recognising that a supermarket is a business and cannot act entirely charitably (34, 48).

An additional challenge noted by our respondents was the uncertainty regarding the performance of healthier food interventions in terms of sales, where unexpected intervention results tended to be attributed to the complexity of the supermarket environment. Specifically, the nutritionists reported that health interventions often ran alongside other marketing interventions in the store which resulted in competing messaging for the customer. This finding aligns with those from a process evaluation of supermarket staff in the Eat Well @ IGA trial (a 12-month randomised controlled trial to increase the healthiness of customer baskets and sales of healthy food in Australia) where it was noted that multiple contextual factors (e.g., traditional business operations focussed on sales/profitability) in the supermarket were found to influence intervention implementation (47). Therefore, there needs to be an appreciation when conducting heathier food interventions for the complexity of the supermarket environment. Indeed, this supposition is closely aligned with another theme we identified that related to supermarket intervention evaluation. At present, supermarket nutritionists’ perspectives of their retailer’s approach to intervention evaluations are mainly light-touch and do not address the complexities of the real supermarket environment. Therefore, this suggests that there should be a shift towards practice-based evidence (49), where the complexities of the supermarket environment are considered when designing, implementing, and evaluating healthy food retail.

Given that the respondents in the current study were aligned on their vision for enabling customer health, it is conceivable that restructuring the supermarket environment so that it is more synergistic (i.e., complementary) in its health messaging may better support customers to purchase healthier food. Indeed, previous literature into healthy choice architecture in supermarkets has found promising results (50). By doing so, this may then prevent unexpected health intervention outcomes (e.g., reduction in product sales), which are ultimately costly to the business. Indeed, emerging findings from the FIO Food project suggest that this is a common complaint from PLWO and FI who report that their shopping experiences are overwhelming with many different contradictory messages (i.e., promotions on less healthy food), and food aisles being constantly reorganised, which makes it difficult to purchase healthier food (25, 36). Similarly, Gustafson et al. (51) suggested that there are cognitive demands placed on shoppers when shopping in-store for healthier food, and for those shoppers who are living on a low income, these demands are greater due to financial constraints. Therefore, the lived experience appears to corroborate the concerns held by the supermarket retailers with regards to competing messaging in store.

Many respondents also highlighted the lack of control they perceived they held over products which were not from their own brand. Own brand products typically include fruit and vegetables, meaning that supermarkets can more easily manipulate promotional activity (albeit funded by the retailer themselves) and the allocated shelf space given to the product (52). However, large, established suppliers who supply well-known household food brands, of which many can be considered HFSS (53), are able to negotiate promotional activity and the allocated shelf space of their products (54, 55), particularly during seasons and key events throughout the year (e.g., Easter, Halloween) (53). Therefore, for example, it is conceivable that discounting of branded confectionary that have been negotiated could be at odds (in terms of promoting a healthier diet) with the promotions of own brand fruit and vegetables, especially given that fruit and vegetable promotions are funded by the retailer rather than suppliers who notoriously have larger promotional budgets (53). Thus, due to consumer demand for certain branded products that is reinforced by complex competitive arrangements between supermarkets and suppliers, it is often not commercially viable to restrict the supply or promotion of branded goods in supermarkets (56). Furthermore, it is imperative that academics and policy makers consider the extent to which supermarkets are culpable for the products that are on promotion. There is a complex commercial food system that needs to be considered if we are to create a healthier and more sustainable supermarket environment, which is also commercially viable (57). Indeed, when governments have imposed mandatory regulations on suppliers, such as in the UK with the Soft Drinks Industry Levy, which taxed manufacturers who did not reformulate their beverages to reduce the sugar content, this very different approach has resulted in promising results by significantly reducing sugar intake in children and adults (58).

The supermarket nutritionists in our study highlighted that there was a perceived intention-behaviour gap in customers’ healthy food purchasing. Specifically, when health interventions were implemented based reducing prices of healthier food, there was not the expected increase in sales of the promoted product. This finding is in line with Martinez et al. (32), where retailers located in low-income areas indicated that whilst price was their customers’ greatest barrier to purchasing healthier items, there was no guarantee that implementing price reductions would result in an increase in sales. Instead, they therefore inferred (rightly or wrongly) that the main driver of purchasing behaviour was not price but instead demand—believing that customers did not ultimately want the healthier food. Indeed, while this observation could be explained by it reflecting customers’ food preferences and the risk of food waste associated with perishable, healthier food, especially in families with children (59), it could equally reflect the barriers encountered from the food environment, such as convenience, time, and space to prepare (60). Similarly, Kim et al. (52) reported that retailers were hesitant to stock healthier foods as they were considered a ‘high-risk investment’ as by doing so can result in loss of sales. Therefore, this suggests that health interventions that operate using price levers to promote healthier food are not the panacea. Instead, more complex, multi-lever interventions may be more effective in increasing healthier, more sustainable food sales, which would ultimately benefit both the customer (i.e., customer health) and the retailer (i.e., increased sales). However, to achieve this would require a shift in thinking and working, including long term investment and adequate funding (61). Indeed, in a randomised controlled trial of people living with low income in an area of high obesity prevalence in the United States, employing a health intervention that used promotion (food samples), education (recipe cards), placement (aisle-end cap placement), and price ($1 discount coupon for buying promoted fruit and vegetables) to encourage fruit and vegetable purchasing, they found significant increases in purchasing of these foods (62). Interventions that use multiple levers perhaps are more likely to affect multiple aspects of the customer’s in-store experience and this reinforces the previous point that supermarkets’ in-store environments should be conducive with encouraging healthier, more sustainable food purchasing.

When discussing their approach to health and sustainability interventions, one insight that emerged from respondents was that intervention evaluation varied widely. Some respondents suggested their supermarkets were more advanced with how in-depth their approaches to intervention evaluation were and with how often they conducted intervention evaluation, whereas other respondents mentioned they were actively running interventions without assessing their impact. Vogel et al. (63) synthesised their research teams’ experiences of working with supermarket chains to evaluate strategies to promote healthy eating. They similarly recommended that supermarket intervention research should use more robust study designs, but acknowledged that this would be difficult in commercially, competitive real-world settings. Therefore, there is a clear need for a best practice guide for retailers that provides support and guidance with how to design, conduct, and evaluate health interventions to produce reproducible, transparent findings that can facilitate positive, meaningful change to customers’ purchasing behaviour.

Despite there being challenges with the evaluation of health (and sustainability) interventions, all respondents expressed a desire to work with other retailers and academics to enable knowledge mobilisation in this space. This is complementary with the concept of co-creation (i.e., collaborative approach of creative problem-solving with multiple stakeholders), and previous research has shown that this approach is necessitated in health interventions design in supermarkets (33, 47, 64, 65). Therefore, providing retailers with the appropriate infrastructure to facilitate this collaboration warrants further investigation.

Although the current study was interested in supermarket nutritionists’ experiences of promoting both healthier and sustainable food purchases, most respondents tended to report on their experiences of health interventions rather than sustainability. It became apparent throughout the interviews that this was a result of a lack of a ‘legal definition’ of sustainable food, which appeared to conflate three distinct issues. First, respondents highlighted a definitional issue, noting a lack of a standardised definition for what legally constituted a sustainable food product. Second, respondents highlighted a measurement issue, emphasising how there are no validated metrics for quantifying the sustainability of one product relative to another, unlike in health where there are established metrics such as HFSS status (37) and the multiple traffic light scheme (66). Third, respondents raised communications-law issues, explaining that current marketing and environmental-claims regulations do not provide clear criteria for when sustainability claims are legally permissible and cited examples of violations (67). This regulatory ambiguity heightened perceptions of legal risk, and respondents described the threat of legal proceedings as a major barrier to implementing any interventions on sustainable food. Nevertheless, respondents noted their supermarkets were proactive in their response to the complexity of defining a sustainable product by utilising accredited sourcing (e.g., fair trade) of a product, or by using government dietary recommendations such as the Eatwell Guide, to sensibly recommend a diet that is in favour of health and also sustainability (68). Therefore, this speaks to the need for upstream change (e.g., guardrails for environmental claims or minimum evaluation standards) to support retailers with the promotion of healthier and more sustainable food in supermarkets.

The current study had numerous strengths; particularly, it collected insights from senior supermarket nutritionists from supermarkets who represented 85% of the grocery market share in the UK, therefore providing a relatively representative snapshot of UK supermarket retail sector. However, as is characteristic with thematic analysis, themes reflect patterns of meaning from codes across data. Therefore, if only one code is apparent in one supermarket’s data, it is not likely strong enough to warrant a standalone theme (38). Whilst we were able to collect data from nutritionists from multiple retailers, the discounter supermarkets were under-represented. Thus, as these retail entities operate using a different business model to traditional supermarkets, it would be interesting in future research to explore exclusively the perspectives and experiences of discounter supermarkets in relation to affordability interventions and healthier and more sustainable food promotion. This is because discounters are already offering their products at the lowest possible price, and it is these supermarkets who are often frequented by vulnerable groups (31). Additionally, the current research explored only the perspectives of supermarket nutritionists, which excluded the perspectives of other members in the organisation, such as from the commercial team, or from supermarket store managers, whose perspectives may differ. Nevertheless, this paper advances the existing literature into healthier and more sustainable food promotion in supermarkets by adding the perspective of the retailer, albeit the supermarket nutritionist. This perspective has largely been ignored in previous research despite the pivotal role the food retail sector plays in enabling or constraining individual food purchase choices and the proactive role this can play in addressing dietary inequalities.

## 5 Conclusion

The current study has illuminated how the implementation of affordability interventions to support customers to purchase healthier and more sustainable food (mainly healthier food) requires the retailer to consider multiple multifaceted challenges. Supermarkets, therefore, are in a paradoxical situation. Despite a genuine commitment to promoting population and planetary health, and a willingness to collaborate with academics and other food retailers to generate meaningful interventions to support customers, their efforts are often hindered by financial, logistical, commercial and behavioural challenges. These findings underscore the need for a more nuanced understanding of the retail environment and calls for collaborative efforts to create viable interventions that align population and planetary health objectives with economic realities. To achieve substantial and lasting change, our research suggests there is a compelling need for upstream intervention which mandates and supports interventions that use multiple levers including but not limited to addressing affordability. Policy measures and regulatory frameworks should be designed to incentivise healthier, and more sustainable retail which also mitigate the financial risks associated with affordability interventions.

## Data Availability

The datasets presented in this article are not readily available because requests for access are required to be reviewed on a case-by-case basis. Such requests should be directed to Charlotte Hardman, charlotte.hardman@liverpool.ac.uk.
