# Supplementary material for: Supermarket nutritionists’ perspectives, views, and experiences on affordability interventions to support healthier and more environmentally sustainable food purchasing in UK retail settings
Source: Front Nutr. 2025 Dec 16;12:1710661. doi: 10.3389/fnut.2025.1710661 (PMC12750461; doi:10.3389/fnut.2025.1710661)
Supplement: Supplementary file 1 [file Supplementary_file_1.docx]

**Supplementary Files 1**

Interview Schedule

**Introduction to the topic and instructions:**

Hello and welcome to our interview. Thank for agreeing to take part. My name is Dr Rebecca Stone and I am a postdoctoral researcher working at the University of Liverpool on the FIO Food project, I will be facilitating the interview today. Before we start do you have any questions or concerns you’d like to raise that have occurred to you since you agreed to take part?

[Answer any questions]

Super.

So, as you have already heard, the FIO Food project is concerned with helping people living with obesity and food insecurity shop more easily for healthier, more sustainable foods in the supermarket, both online and in-store. We have recently conducted a large online survey with people living with obesity and food insecurity to understand their experiences of shopping for healthy and sustainable food in retail settings. Within this survey we were able to gather some insights into what this group of people thought food retailers could do to enable them to purchase these foods more easily. Therefore, we are interested in understanding your company’s views about health and sustainability, and on the adoption/integration of some/all of the key recommendations obtained from survey.

The interview should last about 30 minutes; however, this depends on how much or how little you feel you have to say. At the end of the interview there will be time for questions if you have any about what you’ve heard throughout the interview. Let me just remind you that I will be recording today’s interview, but your response will be treated with full confidentiality and any potentially identifying information you share, such as names or places, will be removed to ensure anonymity.

Does that all sound ok? Great.

[Answer any questions]

**Interview Questions**

1. How does X decide on/define what a healthy food product is?
2. How does X decide on/define what a sustainable food product is?
3. Do you have a strategy for health and/or sustainability at X?
   - If yes:
     - What is it?
     - Is health or sustainability a priority for X?
     - Who is accountable for delivering the commitments/ strategy on health and sustainability?
       - Board level?
       - Junior level?
     - Do you have targets/ commitments to track progress against your strategy?
     - Does this include influence over your own-label and branded products sold?
     - What happens if you do not meet health or sustainability targets?
       - Is this linked to bonus?
     - How many people in the business do you believe would have health and sustainability measures built into their objectives?
   - If no: why do you think this is?
4. What mechanisms have you used in the past to drive healthier food sales (*strategic level –levers*)
   - How did it work? / how did you evaluate this?

- What went well?
- What could have gone better?
- If resource wasn’t a concern, what trials would you like to trial either because you feel they could be impactful or because there is a gap in the evidence at present?
  - What are the barriers to running these trials today?
- Does X intend to run trials again in future?
  - If Yes – Why / What are the business benefits?
  - If No - Why not?
    - Prompt: Is it funding, consumer ask, business case?

If they haven’t:

- Why have you not? What have been the barriers to these trials?
- Do you have plans to?
- What would enable or prompt X to run interventions in the future?

1. What mechanisms have you used in the past to drive sustainable food sales with consumers (*strategic level –levers*)
   - How did it work? / how did you evaluate this?

- What went well?
- What could have gone better?
- If resource wasn’t a concern, what trials would you like to trial either because you feel they could be impactful or because there is a gap in the evidence at present?
  - What are the barriers to running these trials today?
    - Lack of frameworks?
- Does X intend to run trials again in future?
  - If Yes – Why / What are the business benefits?
  - If No - Why not?
    - Prompt: Is it funding, consumer ask, business case?

If they haven’t:

- Why have you not? What have been the barriers to these trials?
- Do you have plans to?
- What would enable or prompt X to run interventions in the future?

1. As I said at the start, we have conducted a large survey of PLWO and FI. Our respondents indicated that interventions/marketing activations based on price/incentivisation would be most helpful in helping them to purchase healthier food (for example: offers and promotions on healthy food, rewards on supermarket loyalty card when purchasing healthy food, personalised money off promotions).

- What do you think of these interventions/marketing activations?
- Do you think they could work in X?
- If yes – why?
  - Is this based on previous experience?
- If no – why?
  - What are the barriers to doing price?
  - Is this based on previous experience?

1. The survey also indicated that interventions/market activations based on price/incentivisation would be most helpful in helping them to purchase more sustainable food (for example: offers and promotions on sustainable food, rewards on supermarket loyalty card when purchasing sustainable food).

- What do you think of these interventions/market activations?
- Do you think they could work in X?
- If yes – why?
  - Is this based on previous experience?
- If no – why?
  - What are the barriers to doing price?
  - Is this based on previous experience?

1. Do you think there is a profitable mechanism to run interventions on price and incentivisation long term?
   - if so- why?
   - If not – why?

**Concluding the session:**

Great stuff, thanks! Ok, it looks like we’ve come to the end of the interview. Do you have any other questions before we finish?

[Answer any questions]

Don’t worry if you have none right now, you have my email address so please feel free to contact me with anything. Thank you so much again for your time today, it is greatly appreciated.
